# A cross‐sectional observational study of missed nursing care in hospitals in China

**DOI:** 10.1111/jonm.13112

**Published:** 2020-08-17

**Authors:** Hongxia Du, Yuanyuan Yang, Xiaohong Wang, Yuli Zang

**Affiliations:** ^1^ Department of Nursing Jinan Central Hospital Cheeloo College of Medicine Shandong University Jinan Shandong Province China; ^2^ School of Nursing Cheeloo College of Nursing Shandong University Jinan Shandong Province China; ^3^ The Nethersole School of Nursing Faculty of Medicine Chinese University of Hong Kong Shatin, NT Hong Kong SAR China

**Keywords:** medical errors, missed nursing care, nurses, patient safety, risk management

## Abstract

**Aim:**

To identify the risk of missed nursing care (MNC), and contributing factors, in Chinese hospitals.

**Background:**

National reporting of adverse incidents diminishes errors of commission. To further improve service quality and patient safety, MNC should be reduced.

**Methods:**

An online survey comprising the MISSCARE Survey and the McCloskey/Mueller Satisfaction Scale was conducted with a convenience sample of nurses (*n* = 6,158) in 34 Chinese hospitals.

**Results:**

Participants’ mean age was 30.6 (*SD* = 7.014), and 2.5% were male. The most frequently missed nursing care items were basic care (12.7%–51.8%). The most frequently reported reasons were human resource issues (63.1%–88.2%). Being female, no child, better educated, a manager, permanently employed, no night shift, inadequate friend support and job dissatisfaction influenced the perception of MNC (odds ratio 1.00–4.848).

**Conclusions:**

MNC often occurred in basic care involving informal caregivers or in surge status due to a sudden increase in workload.

**Implications for Nursing Management:**

Nurse managers should prioritize effective measures that target delegation competency and mobilization of nurses for flexible repositioning during need.

## 1 BACKGROUND

Patient safety is a priority in health service delivery, and nurses play a critical role in every stage of direct patient care and adverse incident minimization (WHO, World Health Organization, 2019). The primary nursing‐related threats to patient safety are errors of commission and missed nursing care (MNC)—an omission error (Kalisch & Xie, 2014): the former may result in medical errors or malpractice, while the latter is often rationalized and, consequently, left unresolved (Jones, Hamilton, & Murry, 2015).

Globally, numerous countries have developed national reporting systems for adverse incidents, following the WHO guidelines (Larizgoitia, Bouesseau, & Kelley, 2013). These reporting mechanisms are anticipated to reduce errors of commission and omission. However, the system in mainland China is flawed and incomplete; little is known about its potential to reduce MNC (Yao, Kang, Wang, Zhou, & Gong, 2018).

### 1.1 Conceptualization of MNC

**FIGURE 1:**
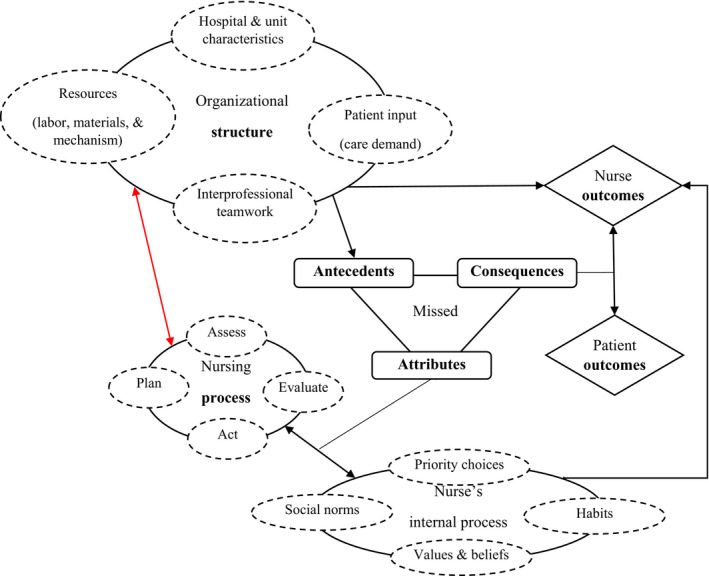
Conceptualization of missed nursing care within Donabedian's quality model

Kalisch (2006) pioneered the investigation of MNC in a qualitative inquiry about regularly missed medical–surgical care and associated reasons. MNC (Figure 1) has been validated regarding its antecedents (e.g. care demand, labour or material allocation, and communication), attributes (e.g. omission of required care because of individual nurse's internal working mechanism: underpinned by values and beliefs, team norms, priority decisions and habits), and consequences or patient outcomes (Kalisch, Landstrom, & Hinshaw, 2009).

According to Donabedian's theory, organisational and human characteristics are interwoven with the structure (hospital and unit level), process (nursing and interprofessional) and outcome components (patients, nurses and systems) of quality health care (Ayanian & Markel, 2016; Kalisch & Xie, 2014). Both patients and nurses benefit from positive processes in the service delivery system. Conversely, negative processes can jeopardize health care systems, threaten individual health and create other detrimental outcomes (e.g. patient falls, health complications, mortality and psychological distress, or nurses’ dissatisfaction) (Ball et al., 2018; Kalisch & Xie, 2014). The nurses integrate structural, process and outcome components of health services for optimal patient outcomes. They are also expected to maximize patient benefit and prevent harm through assessment, care planning, monitoring and surveilling, double‐checking, assistance and interprofessional collaboration (Blackman et al., 2018; Vaismoradi, Tella, Logan, Khakurel, & Vizcaya‐Moreno, 2020).

### 1.2 MNC occurrence and reasons

Inadequate staffing is the most widely examined, and most frequently reported, significant causes of MNC (Ball et al., 2018; Cho, Kim, Yeon, You, & Lee, 2015; Griffiths et al., 2018; Kalisch, Doumit, Lee, & Zein, 2013; Tubbs‐Cooley, Mara, Carle, Mark, & Pickler, 2019). Inadequate staffing increases MNC, while increased MNC often indicates staff shortage. A survey of 300 hospital nurses across nine European countries revealed that MNC and understaffing significantly increased the 30‐day mortality rate and post‐surgical admissions (Ball et al., 2018). In China, nurse staffing is improving, but the shortage of nurses remains the primary concern (Shen et al., 2020). Indeed, inadequate staffing was the most commonly cited significant contributor to MNC (Griffiths et al., 2018).

Individual and organisational characteristics affected MNC occurrence. A study of 864 Icelandic nurses found that nursing teamwork explained an additional 14% MNC variance when unit, role (registered nurse [RN] vs. practical nurse), age and staffing adequacy were controlled (Bragadóttir, Kalisch, & Tryggvadóttir, 2017). A prospective study showed that perceived heavy workload worsened MNC in the neonatal ICU; these cases of MNC involved infection prevention (for invasive treatment), double‐checking, six rights for medication administration, oral feeding and parental involvement (Tubbs‐Cooley et al., 2019).

Accountability and confidence in delegation are the most recent factors found to be linked with MNC. A core value that received advocacy for generations (Krautscheid, 2014), accountability encompasses nurses’ underlying beliefs and values regarding the internal processing of MNC, decision‐making priorities and habit consolidation. The shared accountability among nurses in the same unit (i.e. ward accountability) positively affected MNC. A study of 172 focal nurses (committed MNC) and 123 incoming nurses in Israel showed that personal accountability decreased MNC, while ward accountability had a moderating effect on MNC in those with low personal accountability (Srulovici & Drach‐Zahavy, 2017).

The initial study on MNC engaged RNs and nursing assistants (Kalisch, 2006), so the influence of the scope of practice and job responsibilities warrants more investigation. Saqer, Rub, and R. F. (2018) examined this influence among 362 RNs in Jordan, but no significant correlation was detected between MNC and delegation competency. Nevertheless, approximately 45% MNC variance was explained by nurses’ background (i.e. sex, age, teamwork style and mixed‐shift schedule), while major reasons for MNC were related to labour and material resources. More studies are required to investigate the effect that delegation has on RNs and other caregivers (e.g. pre‐registered nurses, nurse aides and family/paid caregivers).

Unlike in other countries, the involvement of family/paid caregivers for basic care was common in China. Caring for one's vulnerable family members is viewed as an obligation; some trace this perspective to traditional Chinese beliefs concerning family responsibilities and harmonious relationships (Hui, Wenqin, & Yan, 2013; Liang et al., 2018). Generally, nurses reminded family/paid caregivers to execute basic care, which was often associated with genuine and committed family support. Paid caregivers affiliated with companies that had contractual agreements with the hospitals without explicating that nurses took full responsibility for paid caregivers’ performance. It was patients or families who decided whom to be employed (Liang et al., 2018). Many family/paid caregivers cannot follow nurses’ guidance, yet nurses rarely intervened in such cases, and this ultimately led to MNC.

### 1.3 Knowledge gap

Despite the increased attention to MNC, little knowledge was gleaned from interventional studies (Fitzpatrick, 2018). The exceptions to this statement were a train‐the‐trainer study (Kalisch, Xie, & Ronis, 2013) and a study about the effect of a primary nursing model (Moura et al., 2019). The quasi‐experimental study revealed the causal relationship between improved teamwork and reduced MNC among 238 medical–surgical nurses (RNs, licensed practical nurses and nursing assistants) (Kalisch, Xie, et al., 2013). The predictive correlational study detected positive effects of the primary nursing model on MNC, demonstrating that MNC was a good indicator of changes to nursing process, organisation model and accountability (Moura et al., 2019).

The growth of MNC research worldwide did not yield much concerning MNC in China, as existing studies were limited to 500–740 nurses from between two and four tertiary hospitals (Chen et al., 2011; Chen, Liu, & Li, 2015; Si & Qian, 2017). Moreover, there was a noticeable paucity of research about MNC in relation to family/paid caregivers.

### 1.4 Aims

This study aimed to investigate the perceived occurrence of, and reasons for, MNC in hospitals in mainland China. Research hypotheses include the following: (a) MNC is more common when nursing work is shared with family/ paid caregivers; (b) demographic and organisational characteristics influence the perception of MNC; (c) higher job satisfaction is associated with less MNC; and (d) human resource challenges are frequently reported as main reason for MNC.

## 2 METHODS

A cross‐sectional observational design was employed in the online survey of RNs at hospitals in a coastal province's capital city (approximate population: 8.7 million; area: 10, 244 km^2^).

### 2.1 Sampling and participants

Using convenience sampling (participant details in Table 1), all tertiary and secondary hospitals under the governance of the city's health commission were involved, to ensure the contextual similarity between hospitals. Furthermore, all front‐line RNs were eligible to participate. Those excluded were either full‐time student interns, being trained elsewhere or on maternity/sick leave. Persons with a history of mental disorders were disqualified, given their potential negative association with MNC. In this study, individuals without Internet access, computers or mobile devices were considered as disqualified.

**TABLE 1:** Participants’ demographic characteristics (*N* = 6,158)

Groups	Participants		Non‐parametric test^a^	
	*n*	%	Statistics	*^p^*
Age group (years)				
<25	1,185	19.2	198.724	<.001
25–30	2,575	41.8		
31–35	1,132	18.4		
>35	1,266	20.6		
Sex				
Female	6,003	97.5	−4.156	<.001
Male	155	2.5		
Marital status				
Married	4,346	70.6	130.365	<.001
Single	1,744	28.3		
Divorced and others	68	1.1		
Children				
None	2,374	38.6	138.300	<.001
One	2,717	44.1		
Two or more	1,067	17.3		
Education				
Below bachelor's degree	1,676	27.2	−9.919	<.001
Bachelor's/master's degree	4,482	72.8		
Employment				
Contract‐based	4,780	77.6	−12.702	<.001
Permanent	1,378	22.4		
Clinical instructor				
No	5,016	81.5	−8.803	<.001
Yes	1,142	18.5		
Head nurse				
No	5,628	91.4	−11.749	<.001
Yes	530	8.6		
Technical title				
Nurse	2,161	35.1	230.637	<.001
Teaching nurse	2,362	38.4		
Attending nurse	1,535	24.9		
(Association) chief nurse	100	1.6		
Years of work				
<5	2,157	35.0	174.440	<.001
5–10	1,968	32.0		
10–20	1,321	21.5		
≥20	712	11.6		
Type of working hospital				
Tertiary general	1,455	23.6	65.786	<.001
Tertiary specialty	1,010	16.4		
Secondary general	3,077	50.0		
Secondary specialty	616	10.0		
Night shift/month (*n*)				
0	1,591	25.8	69.030	<.001
1–4	1,323	21.5		
5–9	2,907	47.2		
>10	337	5.5		
Overtime in the last 3 months (hours)				
0	2,597	42.2	103.016	<.001
1–12	2,344	38.1		
>12	1,217	19.8		
Big events in the last year^b^				
No	4,591	74.6	−6.693	<.001
Yes	1,567	25.4		
Support from family				
Inadequate	671	10.9	−7.931	<.001
Adequate	5,487	89.1		
Support from friends				
Inadequate	1,010	16.4	−9.754	<.001
Adequate	5,148	83.6		
Intention to resign				
No	5,687	92.4	−7.628	<.001
Yes	471	7.6		
Job satisfaction^c^				
Dissatisfied	604	9.8	292.586	<.001
Average	2,478	40.2		
Satisfied	3,076	50.0		

^a^ Mann–Whitney *U* test (two‐group comparison); Kruskal–Wallis *H* test (≥ 3‐group comparison).

^b^ for example bereavement, hospitalization, divorce and change of residence.

^c^ Reclassified from the 31st item of the McCloskey/Mueller Satisfaction Scale using a 5‐point scaling scheme.

PASS software (NCSS, Kaysville, Utah) was used to estimate the sample size, by assuming that the highest rate of MNC was higher than the reported (66.16%, 393/594; Chen et al., 2011). The test for the difference between the two Poisson rates was performed to detect 1% difference between MNC rates in tertiary and secondary hospitals. In total, 4,785 valid responses were needed for tertiary (*N* = 594) and secondary (*N* = 4,191) hospitals.

### 2.2 Instrument

The primary outcome was MNC, defined as the failure to accomplish required care, as anticipated (Kalisch & Xie, 2014). Secondary outcomes were reasons for MNC and job satisfaction. The reasons can provide insight into the ways in which nurses explain or justify MNC, while job satisfaction is a significant potential indicator of MNC outcomes (Kalisch et al., 2009; Kalisch & Xie, 2014). The MISSCARE Survey (Kalisch & Williams, 2009) and the McCloskey/Mueller Satisfaction Scale (MMSS) (Mueller & McCloskey, 1990) were used to measure the aforementioned outcome variables.

#### 2.2.1 Background information sheet

In addition to sociodemographic data (e.g. age, ethnicity, sex, years of work, marriage, education and position), clinical job features (e.g. preceptorship, night shifts and overtime) and subjective perceptions (i.e. intention to resign, support from family/friends and stressful events such as bereavement) deemed relevant to MNC were also inquired about.

#### 2.2.2 MISSCARE Survey

The MISSCARE Survey is comprised of Part A (MNC; Table 2) and Part B (reasons for MNC; Table 3). Part A uses a 5‐point Likert scale to measure the frequency (1 = never, 2 = rarely, 3 = occasionally, 4 = frequently and 5 = always) of MNC in one's own unit during the prior week. The total number of items with a score of 4 or 5 is the scale score (0–29), and a higher score indicates more MNC. Part B uses a 4‐point scale (1 = not a reason, 2 = minor, 3 = moderate and 4 = significant reason) to rate reasons for MNC.

**TABLE 2:** Scores of missed nursing care items

Item statement^a^		Risk level^b^	Frequency score^d^		Missed (*N* = 6,158)		
			Mean	*SD*	*n*	%	Rank^e^
Basic 4	Setting up meals for patients who feed themselves	1	1.55	1.68	3,190	51.80	1
Basic 3	Feeding patient when the food is still warm	1^c^	1.51	1.44	2,455	39.87	2
Basic 11	Patient bathing/skin care	1	1.56	1.29	2,322	37.71	3
Basic 1	Ambulation three times per day or as ordered	2	1.37	1.13	2,321	37.69	4
Basic 2	Turning patient every 2 hr	2	1.24	0.91	1,127	18.30	5
Action 10	Emotional support for patient and/or family	2	1.4	0.87	1,109	18.01	6
Action 5	Medications administered within 30 min before or after scheduled time	1^c^	1.4	0.93	971	15.77	7
Action 25	Assist with toileting needs within 5 min of request	2	1.39	0.86	782	12.70	8
Basic 12	Mouth care	1^c^	1.22	0.93	780	12.67	9
***Plan 24***	***Attend medical conference and/or nursing round report***	1 ^c^	1.31	0.79	714	11.59	10
***Plan 23***	***Communicate with doctors about patients’ condition***	1	1.52	0.74	671	10.90	11
***Plan 16***	Patient discharge planning and teaching ***involving family caregivers***	1	1.31	0.75	649	10.54	12
Hand 15	Handwashing	2	1.2	0.72	643	10.44	13
***Action 14***	***Health education for knowledge of disease***	2	1.22	0.72	576	9.35	14
Assess 18	Patient assessments performed each shift	1	1.39	0.72	571	9.27	15
***Assess 19***	***Reassessments*** according to patients’ condition	1	1.7	0.67	482	7.83	16
***Action 28***	***Pain relief measures/pain care***	1	2.91	0.67	436	7.08	17
***Assess 22***	Assess ***reactions*** to medications	1	2.43	0.65	430	6.98	18
***Action 26***	***Wound* care**	1	1.48	0.7	430	6.98	19
***Action 9***	***Inform patients of test and investigation procedures and cautious matters***	1	1.46	0.66	423	6.87	20
***Assess 13***	IV/central line site care and assessments according to hospital policy and ***standards***	1	1.71	0.64	359	5.83	21
***Action 21***	***Immediate medical orders* acted on within 15 min, *except for resuscitation or most critical care***	1	2.27	0.61	333	5.41	22
Action 20	Response to call light is initiated within 5 min	2	1.44	0.59	330	5.36	23
Assess 8	Full documentation of all necessary data	1 ^c^	1.21	0.57	285	4.63	24
***Action 27***	***Drainage catheter care***	1	2.2	0.54	215	3.49	25
Assess 7	Monitoring intake/output	2	1.32	0.52	209	3.39	26
Assess 17	Bedside glucose monitoring as ordered	1	1.32	0.56	202	3.28	27
***Action 29***	***Patient sample collection as required***	1 ^c^	1.62	0.51	194	3.15	28
Assess 6	Vital signs assessed as ordered	1 ^c^	1.44	0.5	185	3.00	29

Abbreviations: Action, nursing intervention; Assess, nursing assessment; Basic, basic care; Hand, handwashing; IV, intravenous; Plan, care plan; *SD*, standard deviation.

^a^ Modified (italic) or added items (underlined italic).

^b^ Risk of harmful consequences from ‘1 = no/little harm’ to ‘2 = moderate/severe harm’.

^c^ Rated as ‘2 = moderate/severe harm to patients’ for those in resuscitation or with other critical conditions.

^d^ Mean and *SD* of item scores from ‘1 = never missed’ to ‘5 = always missed’.

^e^ Order from the highest to lowest percentage of items rated as ‘occasionally/frequently/always missed’.

**TABLE 3:** Scores of reasons for missed nursing care

Item number and statement^a^		Reason score^b^		Reason (*N* = 6,158)		
		Mean	*SD*	*n*	%	Rank^c^
3	Unexpected rise in patient volume and/or acuity on the unit	1.85	1.05	5,433	88.2	1
***2***	***Critically ill patients associated with heavy workload***	1.86	1.05	5,411	87.9	2
1	Inadequate number of staff	1.9	1.082	5,331	86.6	3
***22***	***Patient's and family's refusal***	2.32	1.09	5,005	81.3	4
***4***	***Inadequate number of assistive and/or clerical personnel***	2.41	1.071	4,880	79.2	5
***16***	***Heavy admission and discharge activity***	2.5	1.13	4,567	74.2	6
5	Unbalanced patient assignments	2.6	1.077	4,514	73.3	7
***17***	***Underdeveloped management and quality assurance system***	2.53	1.14	4,456	72.4	8
9	Supplies/equipment not available when needed	2.63	1.11	4,369	70.9	9
8	Other departments did not provide the care needed (e.g. physical therapy did not ambulate the patient)	2.7	1.06	4,365	70.9	10
6	Medications were not available when needed	2.65	1.13	4,250	69.0	11
***7***	Inadequate hand‐off from previous shift or sending unit ***(previous shift/unit failed to take good care of patients)***	2.75	1.09	4,153	67.4	12
12	Tension or communication breakdowns with other ancillary/support departments	2.79	1.06	4,104	66.6	13
10	Supplies/equipment not functioning properly when needed	2.72	1.13	4,096	66.5	14
11	Lack of back‐up support from team members	2.83	1.04	4,063	66.0	15
***21***	***Nurses with a weak ability (observation, planning, flexible coping, etc.)***	2.75	1.13	4,005	65.0	16
***18***	***Underdeveloped role description/workflow***	2.78	1.1	3,971	64.5	17
***20***	***Nurses with less comprehensive knowledge***	2.84	1.09	3,886	63.1	18
***19***	***Nurses with less job responsibility***	2.8	1.18	3,684	59.8	19
14	Tension or communication breakdowns with the medical staff	2.96	1.08	3,537	57.4	20
13	Tension or communication breakdowns within the nursing team	3.04	1.06	3,354	54.5	21
***15***	***Nurses did non‐nursing work***	2.93	1.16	3,334	54.1	22

Abbreviations: *SD*, standard deviation.

^a^ Modified (italic) or added items (underlined italic).

^b^ Mean and *SD* of item scores from ‘1 = not a reason’ to ‘4 = major reason’.

^c^ Order from the highest to lowest percentage of items rated as ‘minor/moderate/major reason’.

The earliest Chinese version of the MISSCARE Survey was selected due to its semantic equivalence to the original (Kalisch, Tschannen, Lee, & Friese, 2011), practice suitability and linguistic propriety. It contains modified or added items (e.g. patient/family refusal; Tables 2, 3). These changes were made by Chinese developers, through expert consultation, to reflect the scope and context of local practices (Chen et al., 2011), for instance patient/family refusal for prescribed treatments. The Chinese version reached a content validity index (CVI) of 0.74 and internal reliability (Cronbach's alpha, α) of 0.84, while this study showed a higher α of 0.90.

Nurses’ perception of risk influenced their responses. Thus, a panel of three senior head nurses was organised to rate the risk of harmful consequences of MNC. Using a 4‐point scale, responses ranged from 1 (none), 2 (mild), 3 (moderate), to 4 (severe harm). The rating of 1 or 2 was scored as 0 (no/low risk), while that of 3 or 4 was scored as 1 (risky). The kappa of 0.308 suggested a fair inter‐rater agreement between experts, according to the criteria of 0.21–0.40 (Landis & Koch, 1977). Items that were disagreed upon (17/29, 58.6%) were discussed to arrive at a solution.

#### 2.2.3 McCloskey/Mueller Satisfaction Scale

The MMSS (Mueller & McCloskey, 1990) is a globally employed tool that measures job satisfaction. A 5‐point scale ranging from 1 (‘very dissatisfied’) to 5 (‘very satisfied’) was used for dimensions about work conditions and supervisor support, scheduling, social and interaction opportunities, collegial relationships and support, scholarly opportunities, salary and benefits, and support for family responsibilities. The sum of item scores (i.e. scale score) ranged from 30 to 150, with a higher score indicating greater job satisfaction. Zheng (2009) adapted the original MMSS to reflect Chinese nursing practice. The item of ‘opportunities for part‐time work’ was removed, given that few nurses had part‐time jobs. This resulted in the 30‐item scale (Table 2) with high CVI (0.94) and α (0.95) approximate to that of this study (α = 0.97).

### 2.3 Data collection

An online survey with a sharable hyperlink was created through the Wenjuanxing Web‐based platform (Ranxing, Changsha, China). The platform integrated the information and consent sheet, background sheet, MISSCARE Survey and MMSS. Between 6 September and 15 October 2018, the first author called hospital nursing directors to explain the study's background, purpose, data collection methods and ethical issues. With their approval and coordination, the hyperlink was shared with head nurses and then eligible nurses. Participants received the same information that was shared with nursing directors. When all questions were answered satisfactorily, the participants e‐signed the consent form and completed the survey. Upon the submission of answers, the data file was generated and immediately ready for download through the Web platform.

### 2.4 Data analysis

Descriptive and frequency analyses, and correlation, and reliability analyses were performed with SPSS 26.0 (IBM Corp). For the bivariate logistic analysis, the dependent dichotomous variable was scored using the MNC cut‐off score of 3 (approximately 10% of all 29 MNC items with a response of ‘never’ or ‘rarely’ missed). Omission error was 1 if ≥3 items were missed; otherwise, it was 0 (no/little omission error). Background data were added or removed as predictive covariate variables during the backward stepwise analysis (likelihood‐ratio method). As the Kolmogorov–Smirnov test showed that the MNC score was not normally distributed (*p* < .001), the non‐parametric tests (i.e. Mann–Whitney *U* test and Kruskal–Wallis *H* test) were executed to compare the scores between different subgroups. The independent‐sample *t* test was used to compare the missing rate of MNC items engaging family/paid caregivers with that of other items. It was also used to compare the rate of human resource‐related MNC reason items with that of other items.

## 3 RESULTS

A total of 87.9% of eligible nurses (*N* = 6,419) completed the survey in 34 hospitals (86–2,000 beds [mean 517.0, *SD* 391.871]; 65–1,092 nurses [mean 345.6, *SD* 230.082]). Excluding those (4.1%) with extreme or central tendency responses, 95.9% (*N* = 6,158) were valid.

### 3.1 Participants’ characteristics

Participants were 19 to 60 years old (mean 30.56, *SD* 7.014), and 2.5% were male. The majority were married and had worked as contract‐based RNs for ≤10 years. Most had night shifts (74.2%), and 57.9% worked overtime. Few had faced life events in the preceding year.

### 3.2 MNC and associated reasons

47.8% of participants reported 1–27 (mean 2.98, *SD* 2.582) missed items in their units during the past week. Moreover, 5.9% found that their units had > 5 frequently/always missed items. The most frequently (mean 27.17%, *SD* 14.585) indicated items were meal preparation, feeding warm meals, ambulation, bathing, body turning, emotional support, timely medication, toileting and oral care (Table 2). These items were rated as ‘no/low risk’, except oral care and medication for critical cases. Furthermore, the work of family/paid caregivers was more commonly reported as missed than other items (t = 4.161, *p* = .003 < 0.05).

The units reported to have >3 MNC items were mainly from general hospitals (secondary, 46.9%; tertiary, 29.1%). Over 10% of participants in general hospitals reported MNC in cardiac (secondary, 13.1%; tertiary, 7.5%) and neurological (secondary, 12.6%; tertiary, 12.0%) departments. The most frequently reported units with high MNC were neurological (11.5%), cardiac (9.9%) and orthopaedic (8.6%).

On average, 69.9% (*SD* = 0.100) considered all listed reasons as ‘reason’ for MNC. The most highly recognized reasons were largely related to workforce shortages (mean 81.53%, *SD* 6.294; t = 6.255, *p* < .001, compared with other reasons): unexpected increase in patients, critical caseload, nurse shortages, patient/family refusal, inadequate administrative staff, increased discharge/admission and unbalanced caseload. In cases of discharge/admission or family/patient refusal, nurses require extensive communication with stakeholders to resolve concerns, while inappropriately assigned caseload made some nurses too busy—a rectifiable human resource issue.

### 3.3 Factors influencing MNC

All sociodemographic (e.g. marriage and education) and job‐related (e.g. employment, manager position and night shift) factors, as well as some psychological ones (e.g. job satisfaction and family/friend support), significantly affected the reporting of MNC (Table 1). It was more likely (odds ratio [OR]: mean 1.454, *SD* 1.248) for the following participants to report MNC (*β* > 0, *p*s < .05): females, those not working the night shift, those with inadequate friend support and those with low job satisfaction. Participants who reported less (*β* < 0, *p*s < .05) had lower education, were staff nurses, were from lower levels of the hospital, were non‐parents or were non‐permanent employees (Table 4).

**TABLE 4:** Outcomes of the backward stepwise bivariate logistic regression (*N* = 6,158)

Variables	B	*SE*	Wald test			Odds ratio (OR)		
						OR value	95% CI	
			Wald	*df*	*p*		Lower	Upper
Female	0.852	0.376	5.130	1	.024*	2.344	1.121	4.898
Below bachelor's degree education	−0.277	0.108	6.632	1	.010*	0.758	0.614	0.936
Not a head nurse	−0.472	0.137	11.876	1	.001**	0.624	0.477	0.816
Hospital type			10.371	3	.016*			
Tertiary general	−0.129	0.143	0.811	1	.368	0.879	0.665	1.163
Tertiary specialty	−0.458	0.161	8.123	1	.004**	0.632	0.461	0.867
Secondary general	−0.261	0.132	3.926	1	.048*	0.770	0.595	0.997
Contract employment	−0.297	0.106	7.803	1	.005**	0.743	0.604	0.915
Night shift/month (*n*)			21.662	3	<.001**			
0	0.408	0.196	4.349	1	.037*	1.504	1.025	2.209
1–4	0.041	0.197	0.043	1	.835	1.042	0.708	1.534
5–9	−0.083	0.183	0.209	1	.648	0.920	0.643	1.316
Intention to resign	−0.248	0.133	3.482	1	.062	0.781	0.602	1.013
Children (*n*)			33.340	2	<.001**			
0	−0.567	0.123	21.246	1	<.001**	0.567	0.445	0.722
1	0.013	0.100	0.017	1	.897	1.013	0.832	1.233
Inadequate support from friends	0.332	0.099	11.211	1	.001**	1.393	1.147	1.691
Job satisfaction			164.257	2	<.001**			
Dissatisfied	1.579	0.130	147.957	1	<.001**	4.848	3.759	6.252
General	0.869	0.091	91.409	1	<.001**	2.384	1.995	2.848
Constant	−2.162	0.472	21.006	1	<.001**	0.115		

Abbreviations: CI, confidence interval; *SE*, standard error.

* *p* < .05.

** *p* < .01.

### 3.4 MNC and job satisfaction

Participants were moderately satisfied with their job, as demonstrated by a mean MMSS item score of 3.269 (*SD* 0.404). MNC was negatively and fairly correlated with MMSS (*r_s_* = −.280, *p* < .001) and professional opportunity dimension (*r_s_* = −0.320, *p* < .001).

## 4 DISCUSSION

This study detected the perceived occurrence of MNC (mean 1.55, *SD* 0.404) approaching others (mean 1.56, *SD* 0.4) (Kalisch et al., 2011), suggesting a lower occurrence or reporting of missed care. Although family/paid caregivers were routinely involved in basic and psychosocial care (Hui et al., 2013), these were still the most frequently missed items. The findings of this study are distinct in their revelation of the notable frequency of missed emotional support items and family/paid caregiver refusal. Consequently, nursing managers in Chinese hospitals should review the practice of involving informal caregivers in professional services. Moreover, nurses’ accountability (Srulovici & Drach‐Zahavy, 2017) and delegation competency (Saqer et al., 2018) should be strengthened to improve collaboration with informal caregivers and colleagues.

Regarding the reasons for MNC, the most commonly reported reasons were directly or indirectly related to human resources, as hypothesized (i.e. shortage of nurses). This may be due to staffing inadequacy or heavy workloads (e.g. sudden rise in cases or in severe cases, discharge/admission and uneven workload). Surprisingly, 81.3% of participants indicated patient/family refusal as a moderate or major reason for MNC. This phenomenon is rarely investigated, particularly in comparison with life‐saving treatment refusal (Jin & Zhang, 2020). In practice, nurses sought medical assistance when a patient/family refused certain forms of important care (e.g. medication). For other, less crucial forms of care, nurses documented the fact of refusal for shift reports. Notably, there are no specific guidelines that state how nurses should tackle such refusals. Although it is a great challenge to resolve the reasons for MNC, it must be done to advance professional nursing practice in China.

Basic care (e.g. ambulation, body turning and toileting) is very important for patients with neurological (e.g. stroke) or skeletomuscular (e.g. joint replacement) problems, while timely medication administration is key for patients with cardiovascular (e.g. hypertension) or endocrinological (e.g. diabetes) diseases. This may explain why neurological, cardiac and orthopaedic units had the highest MNC. Like others (Bragadóttir et al., 2017), this study found that MNC was much lower in ICUs (2.4%) than in the three aforementioned units. The workload in lower‐level or specialty hospitals was relatively lower, which may explain the impact of hospital types on MNC reporting (Table 4); other kinds of hospitals (except general tertiary) reported less MNC. Thus, it is recommended that more attention be paid to specific units and hospitals to reduce MNC, for better patient outcomes.

Like others (Bragadóttir et al., 2017; Kalisch, Doumit, et al., 2013; Kalisch et al., 2011), this study observed the significant impact of many sociodemographic factors such as age, education and shifts. Participants with certain characteristics were more likely to report or not to report MNC, for example females, those with job dissatisfaction and those echoing the findings of other studies (Duffy, Culp, & Padrutt, 2018; Kalisch et al., 2011). This study was one of very few to reveal the impact of psychosocial factors such as friend support, life events and job satisfaction. Psychosocial wellness greatly facilitates the reduction of MNC, so nursing managers should pay more attention to measures of psychosocial health in nurses.

In summary, hypotheses related to the influence of informal caregivers’ engagement, individual and contextual characteristics, human resources and job satisfaction over MNC were supported by the findings from this study. For comprehensive interpretation, however, study limitations must be addressed.

### 4.1 Limitations

The sample size exceeded estimates, and 84.3% eligible nurses completed the study. Convenience sampling was used instead of random because rosters for participating hospitals were unavailable. Nonetheless, the use of the former limits study findings’ generalizability to the target population.

Also, nursing directors disseminated the survey hyperlink to potential participants, which may have introduced implicit coercion. It was, however, impossible to successfully recruit nurses to report hospital events while circumventing nursing directors. Since we asked about MNC in participants’ units (instead of omissions or delays on the part of the participants themselves), the impact of social desirability should not be so high as to undermine the truthfulness of responses.

To select the most appropriate version of the three Chinese versions of the MISSCARE Survey (Chen et al., 2011; Chen et al., 2015; Si & Qian, 2017), we used subjective judgement instead of an objective approach (e.g. a concurrent test of three versions). This decision may have jeopardized the internal validity of this study. Besides, the use of multiple versions of the MISSCARE Survey makes it difficult to compare the outcomes in the same country.

### 4.2 Implications for nursing management

Medical error is becoming one of the main causes of death, after cancer and cardiovascular disease. MNC precedes medical errors in nursing, so the effective prevention of the former may contribute significantly to the reduction of the latter.

This study revealed that human resource issues were the most frequently reported reason for MNC, which were associated with sudden increases in workload and/or critical cases. Beyond planning for more nurses, it is more practical to mobilize existing workforces, for the immediate solution for challenging situations (e.g. surges).

Flexible scheduling or repositioning of nurses and on‐call staffing may release the reservoir of nurses to support places with urgent or suddenly increased needs, for example during a pandemic, emergency or disaster. This requires specific training and assessment to bank nurses—especially the young, motivated and willing. Qualified trainees could be deployed immediately for urgent or critical care. The identification and regular training of deployable nurses could be integrated with other nursing development efforts, to reduce conflicts of interest or competition for resources.

This study also revealed gaps in basic care where family/paid caregivers are involved. Nurses’ responsibility and competency in delegating, monitoring and supervising family/paid caregivers shall be strengthened. More specific training (e.g. delegation competency) and professional responsibilities should be emphasized. This way, attending nurses can improve their communication with, and supervision and assistance of direct care involving family/paid caregivers to prevent MNC.

## 5 CONCLUSION

The most frequent MNC in China was mainly committed by family/paid caregivers. Many personal and organisational characteristics influenced the reporting of MNC. The most frequently cited reason for this was workload‐related human resource issues. To minimize MNC, attention should be paid to basic care activities when informal caregivers are involved, as well as nurses with particular characteristics. Increasing the surge capacity or improving the nurses’ delegation competency might effectively minimize MNC; this requires further investigation.

## ETHICAL APPROVAL

The Hospital Medical Ethics Committee (No. 2018‐106‐01) approved this study according to the principles of the Declaration of Helsinki. The original developers approved the use of the existing scales. All nursing directors and eligible nurses were informed of the study and the ethical principles that centred human rights protection and the minimization of harm (e.g. benefit vs. harm, voluntary participation, withdrawal, anonymity and confidentiality). Two authors answered enquires when they were made. All data were saved in password‐protected computers, to be destroyed five years later. No one but the research team can access these data.
